# Differentiation of Adsorptive and Viscous Effects of Dietary Fibres on Bile Acid Release by Means of In Vitro Digestion and Dialysis

**DOI:** 10.3390/ijms19082193

**Published:** 2018-07-27

**Authors:** Susanne Naumann, Ute Schweiggert-Weisz, Stephanie Bader-Mittermaier, Dirk Haller, Peter Eisner

**Affiliations:** 1 ZIEL-Institute for Food & Health, TUM School of Life Sciences Weihenstephan, Technical University of Munich, 85354 Freising, Germany; dirk.haller@tum.de (D.H.); peter.eisner@ivv.fraunhofer.de (P.E.); 2 Fraunhofer Institute for Process Engineering and Packaging (IVV), 85354 Freising, Germany; ute.weisz@ivv.fraunhofer.de (U.S.-W.); stephanie.mittermaier@ivv.fraunhofer.de (S.B.-M.); 3 Chair of Nutrition and Immunology, TUM School of Life Sciences Weihenstephan, Technical University of Munich, 85354 Freising, Germany

**Keywords:** cholesterol, bile acid binding, bile acid excretion, centrifugation, dialysis, diffusion kinetics, barley fibre, β-glucan, citrus fibre, lupin kernel fibre, potato fibre

## Abstract

To explain the cholesterol-reducing effects of dietary fibres, one of the major mechanisms proposed is the reduced reabsorption of bile acids in the ileum. The interaction of dietary fibres with bile acids is associated with their viscous or adsorptive effects. Since these fibre characteristics are difficult to investigate in vivo, suitable in vitro methodologies can contribute to understanding the mechanistic principles. We compared the commonly used centrifugal approach with a modified dialysis method using dietary fibre-rich materials from different sources (i.e., barley, citrus, lupin, and potato). Digestion was simulated in vitro with oral, gastric, and small intestinal digestion environments. The chyme was dialysed and released bile acids were analysed by high-performance liquid chromatography. The centrifugation method showed adsorptive effects only for cholestyramine (reference material) and a high-fibre barley product (1.4 µmol taurocholic acid/100 mg dry matter). Alternatively, the dialysis approach showed higher values of bile acid adsorption (2.3 µmol taurocholic acid/100 mg dry matter) for the high-fibre barley product. This indicated an underestimated adsorption when using the centrifugation method. The results also confirmed that the dialysis method can be used to understand the influence of viscosity on bile acid release. This may be due to entrapment of bile acids in the viscous chyme matrix. Further studies on fibre structure and mechanisms responsible for viscous effects are required to understand the formation of entangled networks responsible for the entrapment of the bile acids.

## 1. Introduction

Hypercholesterolemia is one of the major risk factors for coronary heart disease—the most frequent cardiovascular disease in developed and developing countries [1]. With regard to the protective effect of a healthy and balanced nutrition, the adequate intake of dietary fibres is of particular importance. Threapleton et al. [2] have demonstrated the inverse correlation between coronary risk factors and an increased dietary fibre intake.

While a correlation of cholesterol-lowering effects and the structural and physiochemical properties of dietary fibres has already been reported, the underlying mechanisms are not yet fully understood [3]. One hypothesis is the impairment of fat and cholesterol absorption and their increased secretion induced by the interaction with dietary fibres or the inhibition of relevant enzymes [4]. Besides the decreased intake of cholesterol, various substances may impact its endogenous synthesis. Due to viscous fibres, the glucose absorption rate decelerates, and therefore the postprandial insulin secretion decreases [5]. As insulin is an activator of a rate-limiting enzyme in cholesterol synthesis, this mechanism could contribute to the reduction of cholesterol [6]. Another hypothesis is related to the bacterial fermentation of dietary fibres to produce short chain fatty acids, which could inhibit the hepatic cholesterol synthesis. 

Evidence for the latter mechanism is still inconsistent; there is broad agreement on the decrease in cholesterol level by a reduced reabsorption of bile acids [3,7]. Bile acids are synthesized in the liver from cholesterol and secreted into the small intestine via the gallbladder. Therein, they contribute to the enzymatic degradation and absorption of fats. In the ileum, about 95% of the bile acids are recycled by reabsorption—a process called enterohepatic circulation. Dietary fibres are reported to interact with the bile acids preventing their reabsorption [3]. Therefore, the bile acids are actively excreted and need to be resynthesized in the liver from cholesterol, which presumably reduces high cholesterol levels [8]. Evidence of this process has already been reported in a variety of in vivo studies, which have shown an excess of faecal bile acid excretion after the consumption of specific dietary fibres [9,10,11].

Until now, the properties and the nature of the interaction between the dietary fibres and bile acids leading to low bile absorption is not fully elucidated. Moreover, these aspects are difficult to investigate in vivo due to the limited access to small intestinal samples and the fermentative alteration of bile acids in the colon. Most studies focussing on possible mechanisms to explain the interaction of dietary fibres with bile acids can be concluded in two possibilities. Either bile acids are directly adsorbed to dietary fibres and/or a viscous network is formed by the dietary fibre polymers thereby restricting the bile acid release [3]. As the term bile acid binding is frequently and erroneously used regardless of the underlying mechanism, it often remains indistinct whether the analysed fibres actually have binding properties by adsorption. 

The comparison of results and mechanisms between in vivo and in vitro are limited by the use of different methods. Most studies on bile acid reabsorption have used centrifugation steps to separate unbound bile acids in the supernatant [12,13,14]. However, centrifugal forces differ significantly from the physiological conditions in the human body. Therefore, bile acid binding measured by centrifugation methods may not be appropriate to take into account the viscosity and matrix effects. In addition, methods differ in fibre concentration, parameters of in vitro digestion, and centrifugation conditions. Other approaches include pressure filtration and dialysis to separate unbound bile acids. Some in vitro studies have already demonstrated the suitability of dialysis to determine bile acid release from simulated chymes [15,16,17,18]. Little account has been taken of whether this approach can reflect adsorption as well as viscous properties of dietary fibres. Moreover, it remains to be shown if these properties can be linked to the water soluble fraction of dietary fibres. 

As dialysis is a diffusion-based approach, the concentration gradient between the in vitro digested chyme and the dialysate at initial conditions of dialysis represents the driving factor of bile acid release. Correspondingly, first-order kinetics can be applied to the analysis of bile acid release across a dialysis membrane. This could enable the evaluation of viscous and adsorptive effects of dietary fibres based on the parameters of diffusion kinetics [16,19]. In the present study, we evaluated this approach by dialysis of in vitro digested chyme containing dietary fibre from different sources (i.e., barley, citrus, lupin, and potato) and studied adsorptive as well as viscous effects under simulating conditions of the small intestine.

## 2. Results

### 2.1. Fibre Composition 

Table 1 represents the soluble dietary fibre (SDF), insoluble dietary fibre (IDF), total dietary fibre (TDF), and a ratio of SDF to TDF. The high-fibre barley product showed a TDF value of 29.2 g/100 g dry matter (DM) while the SDF and IDF were 19.8 g/100 g DM and 9.5 g/100 g DM, respectively. In comparison to the high-fibre barley product, the citrus fibre had differing results for TDF (91.2 g/100 g DM) and an IDF value of 79.8 g/100 g DM. Significant (*p* ≤ 0.05) differences were observed between all the samples as analysed by the Tukey test. The cellulose mainly consisted of IDF (99.1 g/100 g DM), which is consistent with the values reported in the literature [14]. Lupin showed the second highest TDF (83.4 g/100 g DM) with a significantly (*p* ≤ 0.05) lower value of SDF (4.8 g/100 g DM) in comparison with other investigated fibres (except cellulose).

### 2.2. Viscosity

**Figure 1:**
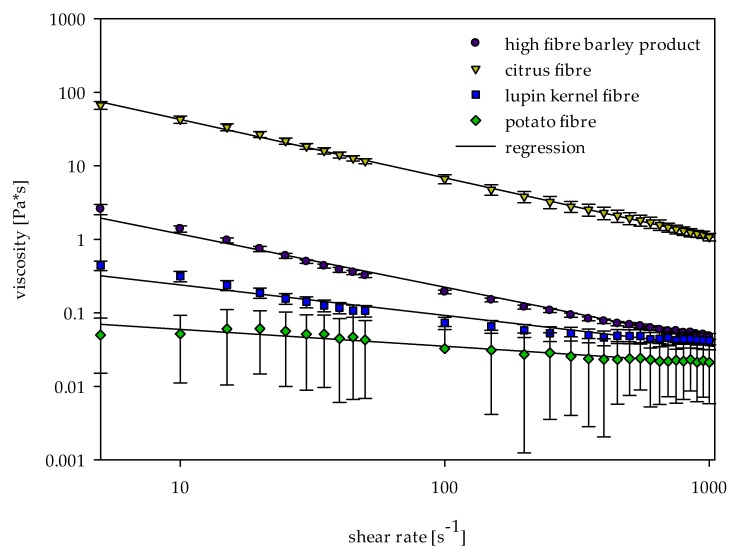
Viscosity of in vitro digested chymes containing different dietary fibre-rich materials as a function of the shear rate (*n* = 3).

The chyme containing citrus fibre was highly viscous in comparison to other selected samples (Figure 1). Interestingly, the viscosity values were surprising due to the smaller amount of SDF in comparison to the high-fibre barley product. The viscosity of the high-fibre barley product was significantly (*p* ≤ 0.05) lower than citrus fibre at all measured shear rates. The viscosity of lupin and potato samples were lower than the above samples, but showed a similar pattern of shear thinning at high shear rates. No definite pattern was observed for the other samples due to low values of viscosity.

### 2.3. In Vitro Determinations of Bile Acid Reabsorption

Centrifugation method was used as a reference method for determination of bile acid reabsorption [12,13,14]. Among the selected dietary fibre-rich materials and references, only the high-fibre barley product and cholestyramine showed bile acid binding properties. The proportion of taurocholic acid bound by the high-fibre barley product was 1.4 ± 0.1 µmol/100 mg DM. Cholestyramine showed bile acid binding of 10.8 ± 0.1 µmol taurocholic acid/100 mg DM. The amount of bile acids bound by cholestyramine was similar to the findings of Kahlon et al. [20] (10.1 ± 0.1 µmol/100 mg DM) and Kim et al. [21] (10.7 ± 0.1 µmol/100 mg DM). Our results for the high-fibre barley product are comparable to the amounts of glycoconjugated bile acids bound by barley flour as reported by Dongowski [12] (1.18–1.55 µmol/100 mg DM). Kahlon and Woodruff [22] reported lower values of bile acid binding for β-glucan enriched barley (0.67 ± 0.02 µmol/100 mg DM), but values (5.3 ± 0.1 µmol/100 mg protein) were similar to our findings (6.0 ± 0.5 µmol/100 mg protein) when compared on equal protein basis. 

**Figure 2:**
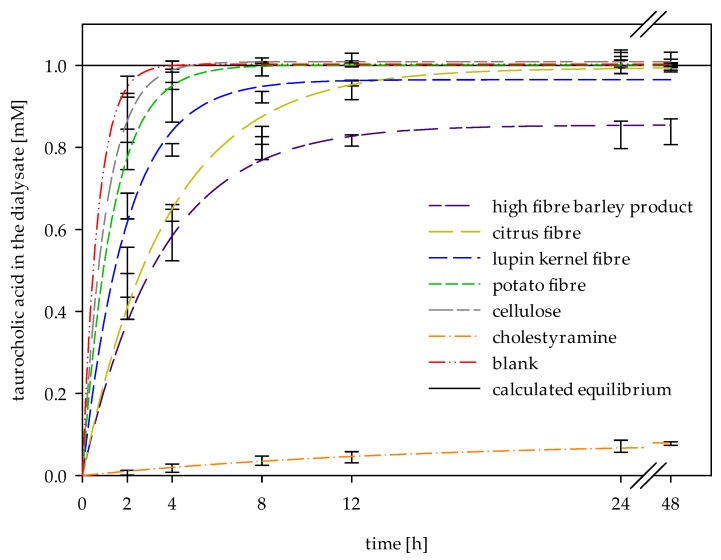
Diffusion kinetics of taurocholic acid release across a dialysis membrane without (blank) and with different dietary fibre-rich materials and references (*n* = 3).

To determine the kinetic of bile acid release using dialysis, the amount of free permeating taurocholic acid was quantified using high-performance liquid chromatography (HPLC). Figure 2 shows the bile acid release curves obtained after in vitro digestion and dialysis of simulated chymes containing different dietary fibre-rich materials, the references (i.e., cellulose and cholestyramine), and the substrate blank. 

The data obtained by triplicate measurements at six dialysis times were fitted using first-order kinetics (Equation (1)).

(1) $$C_{t}=C_{f}\times \left(1-e^{-k\times t}\right)$$

This describes the simplest model for diffusion under dialysis [16,19]. According to Equation (1), the concentration of taurocholic acid after reaching equilibrium (C_f_) and the apparent permeability rate constant (k) were calculated by non-linear regression (Table 2). As can be seen by the correlation coefficients (R²) in Table 2, this model is appropriate to describe the release of bile acids as a function of time.

Without adsorption, the equilibrium concentration (C_f_) of 1 mM can be calculated based on the dilution factor of 10 (in vitro digested chyme diluted with buffer used for dialysis). Adsorptive effects can be recognized at lower C_f_ values, as in the case of reference cholestyramine. The equilibrium concentration in this case was 0.09 mM, which means that 91% of the bile acids were adsorbed. This corresponds to the expectations since cholestyramine is a strong ion exchange resin and forms insoluble complexes with bile acids [23]. The high-fibre barley product adsorbed about 14% of the bile acids. Lupin kernel fibre may have bound about 4% of the bile acid present, though the variability of the results were not within the confidence interval (*p* = 0.25). All other dietary fibre-rich materials did not significantly (*p* ≥ 0.05) deviate from the calculated equilibrium concentration and the negative reference cellulose. 

Although significant (*p* ≤ 0.05) adsorptive effects were only detected for the high-fibre barley product, all other dietary fibres showed a retarded bile acid release from the simulated chymes, as their apparent permeability rate constant k differed significantly (*p* ≤ 0.05) from the blank sample (Figure 2 and Table 2). Cellulose showed a higher rate of bile acid release which was consistent with previous results [12]. Citrus fibre and the high-fibre barley product showed the highest impact on bile acid release, which was more than five times slower than the blank sample. 

### 2.4. Determination of Adsorptive Effects on Bile Acids

**Figure 3:**
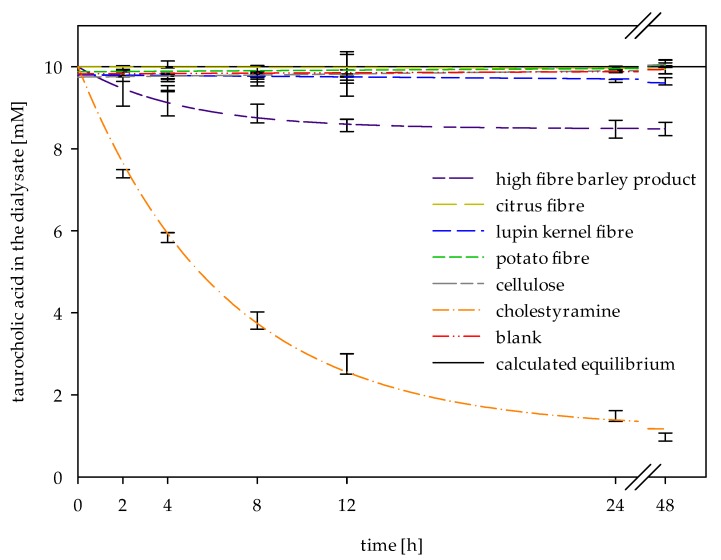
Adsorption of taurocholic acid measured by inverse dialysis model: bile acid concentration as a function of time without (blank) and with different dietary fibre-rich materials and references (*n* = 3).

To confirm the observations of adsorptive effects, an inverse dialysis model for in vitro adsorption was applied (Figure 3). The amount of bile acids adsorbed by the high-fibre barley product (15%) was in accordance with the previous dialysis release kinetics (Table 2). This experiment also clearly displayed the adsorptive potential of cholestyramine, which gradually depleted the bile acid concentration in the dialysate (C_f_ = 1.2 mM, 88% of adsorbed bile acids). These results correspond to the samples that have shown bile acid binding properties using the centrifugation method (high-fibre barley product and cholestyramine). This suggests that only adsorptive effects can be measured with the centrifugation method. 

**Figure 4:**
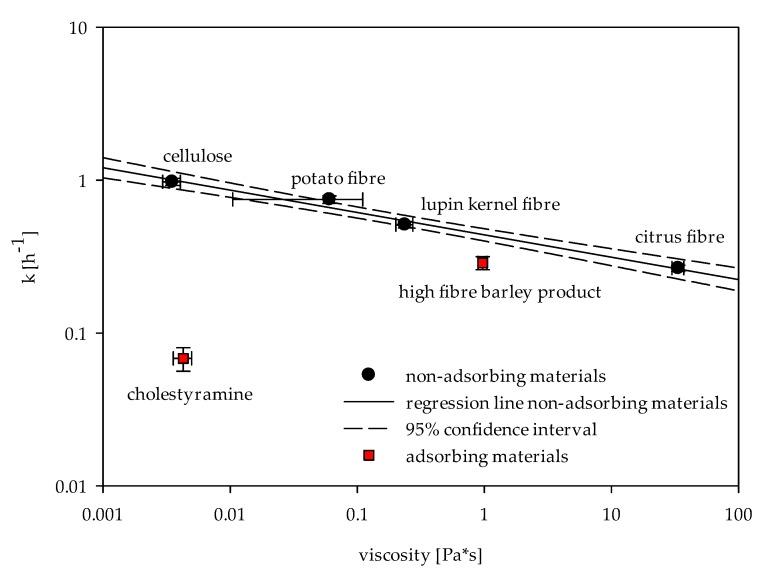
Correlation of apparent permeability rate (k) and the viscosity (shear rate 15 s^−1^) of in vitro digested chymes.

In addition to the concentration gradient between the chyme and the dialysate, the diffusion of bile acids was mainly influenced by the viscosity of the in vitro digested chyme. The impact of adsorptive and viscous effects can be displayed by correlating the apparent permeability rate (k) and the viscosity (Figure 4). Since only small shearing forces occur in the gastrointestinal tract, the correlation was based on the shear viscosity at a shear rate of 15 s^−1^ [16]. 

Without adsorption, the apparent permeability rate (k) correlates with the viscosity of the fibres. For fibres showing adsorptive properties, the impact of adsorption on the release rate was evaluated by the distance to the regression line. Correspondingly, the influence of viscosity on bile acid release in the presence of cholestyramine was minimal. On the other hand, the bile release rate of the sample containing the high-fibre barley product was mainly influenced by viscosity.

## 3. Discussion

Our results support the hypothesis that the influence of dietary fibre-rich materials on the reabsorption of bile acids can be explained as a combination of adsorptive and viscous effects. The impact of both effects has already been discussed based on a variety of in vitro and in vivo findings [3]. In vitro digestion combined with dialysis and kinetic analysis enables the differentiation of both effects, which could be underlined by additional measurements of adsorption and viscosity. 

### 3.1. In Vitro Methodologies

Our results substantiate the suitability of dialysis method for in vitro determination of bile acid release from the simulated chyme. The parameters of first-order kinetics can be linked to the viscosity and bile acid adsorption of fibres. As the concentration gradient decreases with increasing adsorptive effects of dietary fibres, the equilibrium concentration (C_f_) decreases correspondingly. The evaluation of this parameter revealed adsorptive properties of the high-fibre barley product and the reference cholestyramine, which were confirmed by an additional measurement of adsorptive effects via an inverse adsorption experiment (Figure 3). By reducing the concentration of free bile acids present in the in vitro digested chyme, adsorptive effects of dietary fibres also decrease the concentration gradient and thereby influence the apparent release rate (k) [16,19]. This parameter is inversely controlled by the viscosity of the chyme. As the viscosity of the chyme increases, the diffusion and the release rate decreases. Additional viscosity measurements of fibres (e.g., the high-fibre barley product) were helpful to understand the viscous and adsorption effects individually (Figure 4).

Using the common centrifugation method, bile acid binding was detected in chymes containing the high-fibre barley product and cholestyramine. These samples corresponded to the results of adsorptive effects during dialysis experiments. This suggests that by using methods based on centrifugation exclusively adsorptive effects could be determined. The comparison of results also indicated that the adsorptive effects were underestimated by centrifugation: the amount of bile acids bound by the anion exchange resin cholestyramine was 10.8 ± 0.1 µmol taurocholic acid/100 mg DM for centrifugation method; using the dialysis method we detected an adsorption of 13.1 ± 0.1 µmol taurocholic acid/100 mg DM. An even higher deviation between the two methods could be demonstrated for the high-fibre barley product (centrifugation method: 1.4 ± 0.1 µmol taurocholic acid/100 mg DM, dialysis method: 2.3 ± 0.3 µmol taurocholic acid/100 mg DM). 

To ensure the accuracy of the centrifugation method, a complete precipitation of all possibly binding components and constant ratios of supernatants to residues are required. Precipitation is affected by the water solubility of the components, the matrix composition, the dry matter concentration, and the centrifugation conditions [17,24]. Therefore, it remains unclear to what extent soluble fibres and other soluble matrix components are precipitated using centrifugal approaches. Analogously, we observed different residue volumes depending on the sample composition (e.g., residue volume was higher for samples showing high values of IDF (citrus fibre and lupin fibre)). This could explain the underestimation of adsorptive effects using centrifugation in comparison to the dialysis method. In addition, the inconstant deviations of the methods (centrifugation and dialysis) for cholestyramine and the high-fibre barley product could be attributed to different sample compositions. If samples differ in their composition or solubility, conditions for precise measurements by centrifugation method cannot be achieved. The suitability of this method should thus be considered critically, especially for the analysis of soluble fibre.

### 3.2. Evaluation of Dietary Fibre-Rich Materials

Differences between dietary fibre-rich materials from different sources (i.e., barley, citrus, lupin, potato) were assessed using cellulose and cholestyramine. Cellulose is known to have a low bile acid binding capacity [25], which could be confirmed by centrifugation and the in vitro release experiments. Cholestyramine was used as reference for direct binding forces, as it is a strong ion exchange resin adsorbing bile acids [18].

The retardation of bile acid release induced by citrus fibre and potato fibre could be correlated to viscous effects. Soluble fibre content is often described as an indicator for viscous effects caused by soluble fibre components [26,27,28]. However, based on the current data, a correlation between the soluble fibre content and the viscosity of in vitro digested chymes could not be established. Accordingly, despite similar contents of soluble fibre in citrus fibre and potato fibre, the viscosity of the chymes and the bile acid release differed significantly (*p* ≤ 0.05).

Different soluble fibre sources could be associated with different molecular weights and branching patterns. Our results suggest that the soluble fraction of citrus fibre could consist of longer molecules, which are able to form more entanglements, contributing to a higher viscosity of the solution. Depending on the surface area and porosity, insoluble fibres act as filler particles, which could also contribute to the viscosity of the chymes. The particularly high viscous effects of citrus fibre could thus partially be explained by its high insoluble fibre content. This hypothesis is corroborated by the study of Chen et al. [29] which described a high, specific volume of citrus fibre in comparison to other fibre sources. 

Our findings for citrus fibre and potato fibre correspond to previous in vivo studies. The influence of the tested potato fibre on the in vitro bile acid release was comparatively low. Accordingly, there is no indication for cholesterol-lowering of potato fibre by increased bile acid excretion in vivo [30]. The cholesterol-lowering effects of the soluble [31,32,33] and the insoluble fraction [34] of citrus fibre were reported to be associated with an increased faecal bile acid excretion. Our results suggest this effect to be linked with the viscous effects of this fibre. Prospective clinical studies should thus be combined with rheological studies and structure elucidation of this fibre to further elucidate the mechanism of cholesterol reduction.

Our findings suggest that the cholesterol-reduction of the high-fibre barley product could be attributed to the reduced reabsorption of bile acids. Both adsorptive and viscous effects were tested and reported. By correlating the apparent permeability rates and the viscosity of all samples, it could be shown that the release of bile acids in the presence of the high-fibre barley product was mainly influenced by viscosity. Using a similar in vitro dialysis method, Gunness et al. [16] already described the viscous effects of purified barley β-glucan. The viscous effects found in our study may thus be subscribed to the high content of β-glucan in the barley product. The cholesterol-lowering effect of barley β-glucan is well established based on a variety of in vivo studies [35]. Yet, the mechanisms underlying this effect remain to be fully elucidated. Our results substantiate the influence of viscosity on the reduced reabsorption of bile acids by β-glucan-rich fractions. This is in accordance with the in vivo findings of Wolever et al. [36] that demonstrated higher cholesterol-lowering effects of oat β-glucan dependent on its molecular weight, and thus its contribution to viscosity. In contrast, Kim et al. [37] reported the highest values of bile acid binding for oat fractions containing low molecular weight β-glucan. The authors concluded that other components of the oat fraction might also have contributed to the bile acid binding. This is supported by the study of Sayar et al. [38] which described that partial lichenase hydrolysis did not affect bile acid binding of β-glucan-rich oat fractions using an in vitro centrifugation method. These results support our hypothesis that centrifugation-based methods do not reflect the impact of viscosity on the reabsorption of bile acids. The comparability on in vitro centrifugation method with in vivo results is thus greatly impaired.

Referring to the results of previous studies, we assume that the adsorptive effects observed for the high-fibre barley product are not related to its β-glucan content. This is substantiated by the in vitro study of Gunness et al. [16] who reported exclusively viscous effects for purified barley β-glucan on porcine bile acids using an in vitro dialysis method. These results confirmed former nuclear magnetic resonance studies indicating that barley β-glucan does not bind tauroconjugated [39] or glycoconjugated [40] bile acids. Therefore, the contribution of other components must be considered. The adsorptive effects of the barley fraction could be related to insoluble dietary fibre components, which was described for β-glucan enriched barley by Kahlon and Woodruff [22] applying an in vitro centrifugation method. Besides fibre, starch and protein represent the main fractions of barley [41]. Kahlon and Woodruff [42] already described bile acid binding of protein-rich plant materials and also observed the highest relative values of bile acid binding for β-glucan-enriched barley on equal protein basis [22]. A study by Araki et al. [43] described high-binding values for unconjugated bile acids and germinated barley foodstuff. These references indicate a potential role of barley protein in bile acid binding, which should be addressed in further studies. 

Zacherl et al. [17] have already demonstrated a potential influence of lupin kernel fibre on the reabsorption of bile acids in vitro. The bile concentration was assessed in comparison to industrial fibre references after three hours of dialysis. Turnbull et al. [44] similarly concluded that dietary fibre derived from lupin kernels could be suitable as a functional ingredient due to the high-water binding capacity and high induced viscosity during digestion in comparison to other legume sources and negative controls. These results are consistent with the findings of the current study. An in vivo study conducted by Fechner et al. [45] demonstrated that the consumption of lupin kernel fibre contributes to the decrease of blood cholesterol levels in hypercholesterolemic adults. Even though the incorporation of lupin kernel fibre resulted in a slight increase in primary bile acid excretion, the authors hypothesized the formation of short chain fatty acids to be the main contributor to the reduced cholesterol levels. In the current study, the increase in viscosity caused by lupin kernel fibre was low in comparison to the other analysed fibres. Consequently, it could be assumed that the cholesterol reduction found in vivo is caused by several interacting effects.

## 4. Materials and Methods

### 4.1. Chemicals and Enzyme Preparations

Cholestyramine resin (C4650), taurocholic acid sodium salt hydrate (T4009), l-α-lecithin (Egg Yolk, Highly Purified-CAS 8002-43-5), α-amylase from human saliva Type IX-A (A0521), pancreatin from porcine pancreas (P7545), and pepsin from porcine gastric mucosa (P6887) were purchased from Sigma-Aldrich (Saint Louis, MO, USA). All other reagents and chemicals were of analytical grade and supplied by VWR (Darmstadt, Germany).

### 4.2. Dietary Fibre-Rich Materials

A high-fibre barely product SANACEL^®^ betaG containing 19 ± 2% β-glucan (ICC 166) was obtained from CCF GmbH & Co. KG (Gehren, Germany). Citrus fibre Herbacel AQ Citrus-N was kindly provided by Herbafood Ingredients GmbH (Werder, Germany). Lupin kernel fibre (*L. angustifolius* L. Boregine) obtained after de-oiling, extraction, and separation of protein fractions were lyophilized and ground using a Retsch ZM-200 mill (Düsseldorf, Germany) to pass through a 500 µm screen. Lupin seeds were processed on a pilot scale plant based on the method described by D’Agostina, et al. [46]. Cellulose VITACEL^®^ (L 600-30) and potato fibre VITACEL^®^ (KF 200) were supplied by J. Rettenmaier & Soehne GmbH & Co. KG (Rosenberg, Germany).

### 4.3. Fibre Composition

The soluble and insoluble dietary fibre content was determined on an enzymatic–gravimetric basis described in the AOAC Official Method 991.43 [47].

### 4.4. Viscosity

Viscosity of the pre-digested samples was analysed using a rotational rheometer (Physica MCR 301, Anton Paar, Graz, Austria) equipped with RheoPlus software version 3.40 (Anton Paar, Graz, Austria). A parallel plate geometry was selected (diameter: 50 mm, shear gap: 1 mm) and the temperature was kept constant at 37 °C. The samples were pre-sheared at a shear rate of 5 s^−1^ for 20 s and allowed to rest for 20 s before starting the measurement. The viscosity was monitored as a function of the shear rate, which was increased linearly (0–1000 s^−1^).

### 4.5. In Vitro Determination of Bile Acid Reabsorption

The in vitro determination of bile acid reabsorption of chymes containing different fibres was conducted based on a digestion method described by Minekus et al. [24], followed by simulation of bile acid release from the simulated chyme by dialysis or bile acid binding by centrifugation.

#### 4.5.1. In Vitro Digestion

The static in vitro digestion was determined with slight modifications to the methods as described by Minekus et al. [24]. Electrolyte stock solutions (simulated salivary fluid (SSF), simulated gastric fluid (SGF), and simulated intestinal fluid (SIF)) were exactly prepared as published in Minekus et al. [24]. To avoid microbial growth, 0.04% of sodium azide was added to all electrolyte fluids to reach a concentration of 0.02% in the final digestion mixture. The dietary fibre-rich materials and references were weighed in Erlenmeyer flasks and diluted with demineralized water to obtain a final dry matter concentration of 50% (*w*/*w*). For the preparation of substrate blank, the sample volume was substituted by demineralized water. All steps were performed at 37 °C under constant shaking using the water bath G76D (New Brunswick Scientific Co., Edison, NJ, USA) at 150 rpm.

During the oral phase, samples were mixed with SSF (ratio 50:50 (*w*/*w*)) containing α-amylase to achieve 75 IU·mL^−1^ in the final mixture and incubated for 2 min. To simulate the acidic conditions in the stomach, the pH was adjusted to 3 with 1 M HCl. The gastric phase included the addition of SGF (ratio 50:50 (*w*/*w*)) containing pepsin and lecithin to reach a final concentration of 2000 IU·mL^−1^ and 0.17 mM in the digestion mixture and mixing for 2 h. Finally, to simulate the digestion in the small intestine, the pH was neutralised (pH 7) with 1 M NaOH and SIF (ratio 50:50 *w*/*w*) was added, which contained pancreatin to reach 100 IU·mL^−1^ of trypsin activity in the digestion mixture. Taurocholic acid was selected as representative bile acid and used at a concentration of 10 mM [17,24]. The resulting chyme was mixed for 2 h for simulating intestinal digestion. Due to the dilution during the digestion phases, the final chyme resulted in a fibre content of 6.25% dry matter for all samples. 

#### 4.5.2. Bile Acid Binding with Centrifugation Method

**Figure 5:**
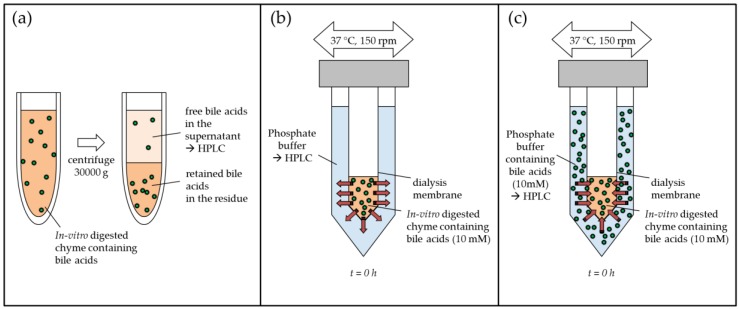
In vitro model approaches for simulation of bile acid reabsorption: (**a**) centrifugation method (Section 4.5.2), (**b**) bile acid release from simulated chyme using dialysis (Section 4.5.3), and (**c**) inverse dialysis model for the determination of adsorptive effects (Section 4.6).

The centrifugation of the pre-digested samples (prepared as described in Section 4.5.1) was based on the method described by Kahlon and Chow [14]. A graphic scheme of the method set-up is presented in Figure 5a. Ten mL of in vitro digested sample material was centrifuged using a Sigma 3 K 30 ultracentrifuge (Sigma Laborzentrifugen GmbH, Osterode am Harz, Germany) at 30,000× *g* and 25 °C for 18 min. The supernatants were decanted and 5 mL of phosphate buffer (0.1 M, pH 6.3) was added, mixed, and centrifuged as before. Supernatants were pooled, diluted by a factor of 10 with demineralized water, filtrated through a 45 µm syringe filter, and analysed for unbound bile acids using HPLC (Agilent, Santa Clara, CA, USA), as described in Section 4.7. Bile acid concentration was calculated and corrected for the mean recovery of bile acids in the substrate blank. 

#### 4.5.3. Bile Acid Release from Simulated Chyme Using Dialysis

4 g of the pre-digested samples (prepared as described in Section 4.5.1), containing 10 mM of taurocholic acid, was transferred into membranes prepared from 16 mm Servapor^®^ 12–14 kDa cut-off dialysis tubings (SERVA Electrophoresis GmbH, Heidelberg, Germany). Dialysis was carried out against 36 mL of 50 mM phosphate buffer (pH 7) containing 0.02% sodium azide using a shaking water bath at 150 rpm and 37 °C. A graphic scheme of the method set-up is presented in Figure 5b. At regular time intervals (2, 4, 8, 12, 24 and 48 h) a 100 μL-aliquot of dialysate was collected and analysed for permeated bile acids by HPLC (Agilent, Santa Clara, CA, USA) as described in Section 4.7.

### 4.6. Inverse Dialysis Model for the Determination of Adsorptive Effects on Bile Acids 

The inverse model for determining adsorptive effects on bile acids based on the methods described in Section 4.5.1 and Section 4.5.3. Analogous 4 g of the pre-digested sample (Section 4.5.1) was filled in the Servapor^®^ tubings and transferred into a flask containing 36 mL of 50 mM phosphate buffer (pH 7) and 0.02% sodium azide. In addition, 10 mM of taurocholic acid was added to the outside buffer (same initial concentration of taurocholic acid on both sides of the dialysis membrane) and the dialysis was conducted in a shaking water bath at 150 rpm and 37 °C. A graphic scheme of the method set-up is presented in Figure 5c. At regular time intervals (2, 4, 8, 12, 24, and 48 h), a 100 μL-aliquot of dialysate was collected and the decrease of taurocholic acid due to potential adsorptive effects were analysed by means of HPLC (Agilent, Santa Clara, CA, USA), as described in Section 4.7.

### 4.7. Bile Acid Quantification Using HPLC

Bile acids were quantified using an Agilent HPLC series 1200 system (Agilent, Santa Clara, CA, USA) equipped with ChemStation software version B.04.02, a G1379 degasser, a G1312B binary gradient pump, a G1367D thermo autosampler, a G1316B column oven, and a G1315C diode array detector. The column used was a NUCLEOSIL^®^ 120-5 C18 (125 × 3.0 mm i.d.; 5 μm particle size) from Macherey–Nagel (Düren, Germany), operated at 40 °C. The mobile phase consisted of 15 mM phosphate buffer (pH 6.5) in water (eluent A) and 15 mM phosphate buffer (pH 6.5) in water and methanol (30:70, *v*/*v*, eluent B). A gradient program was applied as follows: 70% B in the first minute, linearly gradient 70–100% B at 1–3 min, 100% B at 4–7 min, linearly gradient 100–70% B at 8–10 min and hold for 5 min. The injection volume was 10 μL, the flow rate was 0.5 mL/min, and the total run time was 15 min. Taurocholic acid was detected at 200 nm and quantified using a calibration curve. 

### 4.8. Statistical Analysis

Data obtained by triplicate measurements are presented as mean ± standard deviation. The results were evaluated statistically using R version 3.2.4 (https://www.R-project.org) [48]. In all analyses *p* ≤ 0.05 was considered significant. After testing for homogeneity of variance (Bartlett test) and normal distribution (Shapiro–Wilk test), one-way ANOVA with post-hoc Tukey test were performed to separate significant means. Regressions were calculated using SigmaPlot^®^, version 12.5 (Systat Software Inc., San Jose, CA, USA).

## 5. Conclusions

The in vitro model presented in this study describes the measurement of adsorptive and viscous effects on the reduced reabsorption of bile acids. This was confirmed by the correlation of release kinetics with viscosity and adsorption properties. Correspondingly, in accordance to in vivo findings, the release rate in presence of a high-fibre barley product depended mostly on viscous effects, while the adsorption was about 15%. Citrus and lupin kernel fibre preparations, on the other hand, retarded bile acid release mainly by increasing the viscosity of the chyme. Despite its comparably high-soluble fibre content, the apparent release rate was decelerated only slightly in presence of potato fibre. Further experiments confirmed that the soluble dietary fibre content is not suitable as an indicator for bile acid retardation. Using a method based on centrifugation, bile acid binding could primarily be attributed to adsorptive effects. Viscous effects, especially from soluble fibre components, were not represented by this centrifugation method. 

Our findings showed that the reduced reabsorption of bile acids should be assessed holistically to account for interactive effects like the interaction of soluble and insoluble compounds, the impact of porosity, and adsorptive properties. The established method could be further helpful to investigate the mechanisms responsible for cholesterol-reducing effects of dietary fibres. For this purpose, the effects on different bile acids should be studied. 

To link fibre properties and physiological outcomes, clinical studies should be supplemented with structure elucidation and rheological characterization. By differentiating adsorptive and viscous effects the in vitro dialysis method helps to define relevant parameters for prospective clinical studies on cholesterol-lowering effects of dietary fibres. Furthermore, it could act as an initial indicator on components and structures responsible for the reduced reabsorption of bile acids.

To elucidate the mechanisms responsible for viscous effects, further research has to focus on the formation of entangled networks. Further investigations on the structure and composition of dietary fibre-rich materials need to be studied in detail to gain deeper insight into the molecular interactions of dietary fibres and other indigestible food components with bile acids.

## Figures and Tables

**Table 1:** Dietary fibre composition of dietary fibre-rich materials and the reference cellulose (soluble dietary fibre (SDF), insoluble dietary fibre (IDF), total dietary fibre (TDF), *n* = 3).

Sample	TDF (g/100 g DM)	IDF (g/100 g DM)	SDF (g/100 g DM)	SDF/TDF (%)
High-fibre barley product	29.2 ± 1.5 ^a^	9.5 ± 0.2 ^a^	19.8 ± 1.5 ^e^	67.3 ± 1.0 ^e^
Citrus fibre	91.2 ± 0.0 ^d^	79.8 ± 1.2 ^d^	14.9 ± 0.3 ^d^	15.7 ± 0.3 ^c^
Lupin kernel fibre	83.4 ± 0.7 ^c^	78.6 ± 0.4 ^c^	4.8 ± 0.6 ^b^	5.8 ± 0.7 ^b^
Potato fibre	67.8 ± 1.9 ^b^	56.5 ± 0.8 ^b^	11.2 ± 1.7 ^c^	16.5 ± 2.2 ^d^
Cellulose	100.0 ± 0.5 ^e^	99.1 ± 0.2 ^e^	0.8 ± 0.4 ^a^	0.8 ± 0.4 ^a^

Along the column, different letters indicate significant differences on a *p* ≤ 0.05 level basis.

**Table 2:** Correlation coefficients (R^2^), concentration of taurocholic acid after reaching equilibrium (C_f_), and apparent permeability rate constant (k) determined by first-order kinetic fitting of bile acid release from simulated chymes (*n* = 3).

Sample	R^2^	C_f_ (mM)	k (h^−1^)
High-fibre barley product	0.96	0.86 ± 0.02 ^b^	0.29 ± 0.03 ^a,b^
Citrus fibre	0.99	0.99 ± 0.01 ^c^	0.27 ± 0.01 ^a,b^
Lupin kernel fibre	0.99	0.96 ± 0.01 ^c^	0.51 ± 0.03 ^b,c^
Potato fibre	0.99	1.00 ± 0.01 ^c^	0.75 ± 0.04 ^c,d^
Cellulose	1.00	1.01 ± 0.01 ^c^	0.98 ± 0.05 ^d^
Cholestyramine	0.92	0.09 ± 0.01 ^a^	0.07 ± 0.01 ^a^
Blank	1.00	1.00 ± 0.00 ^c^	1.45 ± 0.07 ^e^

Along the column, different letters indicate significant differences on a *p* ≤ 0.05 level basis.

## Abbreviations

DM	dry matter
HPLC	high-performance liquid chromatography
IDF	insoluble dietary fibre
SDF	soluble dietary fibre
SGF	simulated gastric fluid
SIF	simulated intestinal fluid
SSF	simulated salivary fluid
TDF	total dietary fibre
